# NINDS‐supported biospecimen repositories: BioSEND and NHCDR

**DOI:** 10.1002/alz.089661

**Published:** 2025-01-09

**Authors:** Rebecca Price

**Affiliations:** ^1^ National Institute of Neurological Disorders and Stroke, Rockville, MD USA

## Abstract

**Background:**

Access to biospecimens is an oft cited challenge to the progress in research on neurological disorders. Access to clinical biospecimens for development of validated biomarkers and improved cellular models of Alzheimer’s Disease and Alzheimer’s Disease Related Dementias (AD/ADRD) are cited as priorities across several NIH AD/ADRD Research Implementation Milestones (https://www.nia.nih.gov/research/milestones). The National Institute of Neurological Disorders and Stroke (NINDS) is committed to providing high quality biospecimens for NINDS‐mission relevant disorders, including AD/ADRDs.

**Method:**

The NINDS human biospecimen repository, BioSpecimen Exchange for Neurological Disorders (BioSEND), acquires, maintains, and distributes NINDS biospecimen collections for biomarker research in neurological disorders. The NINDS Human Cell and Data Repository (NHCDR) acquires, maintains, and distributes fibroblast, induced pluripotent stem cell (iPSC), and edited iPSC lines from neurological disorders. Samples from BioSEND and NHCDR can be distributed to academic and commercial entities globally to further research from basic to translational stages.

**Result:**

At BioSEND, current biospecimen collections include Parkinson’s Disease, Lewy Body Dementia, Huntington’s Disease, Myalgic Encephalomyelitits/Chronic Fatigue Syndrome, Frontotemporal Dementia, Spinocerebellar Ataxia, and Traumatic Brain Injury. Biospecimen types included DNA, RNA, plasma serum, cerebrospinal fluid (CSF), whole blood, urine, and saliva. At NHCDR, iPSC and fibroblast lines are available from control subjects and patients with various genetic mutations in Alzheimer’s Disease, Amytrophic Lateral Sclerosis, Ataxia‐Telangiectasia, Dystonia, Frontotemporal Dementia, Huntington’s Disease, Lewy Body Dementia, Mild Cognitive Impairment, Myotonic Dystrophy, Parkinson’s Disease, Parkinsonisms, Spinal Muscular Atrophy, Spinal‐Bulbar Muscular Atrophy, and Spinocerebellar Ataxia.

**Conclusion:**

Access to biospecimens is a critical need across all stages of research. The NINDS repositories, BioSEND and NHCDR, provide biofluid and patient‐derived cell lines from various neurological disorders, including AD/ADRDs. Both repositories continue to expand available cohorts which are available to researchers worldwide. More information about BioSEND and available cell lines can be found at https://biosend.org/index.html. More information about NHCDR and available cells, including ordering, can be found at https://stemcells.nindsgenetics.org/.

